# The effect of novel antimicrobial agents on the normal functioning of human intestinal microbiota: a systematic review

**DOI:** 10.3389/fgstr.2023.1159352

**Published:** 2023-08-16

**Authors:** Abayeneh Girma

**Affiliations:** Department of Biology, College of Natural and Computational Science, Mekdela Amba University, Tuluawlia, Ethiopia

**Keywords:** antimicrobial agents, intestinal microbiota, gut microbiota, eubiosis, dysbiosis, probiotics, systematic review

## Abstract

Antimicrobial agents have significant effects on the ecological balance of the human microbiota through incomplete absorption (e.g., orally administered antimicrobial agents) or secretion (e.g., by the salivary glands, in the bile, or from the intestinal mucosa) of the agents. This study aimed to examine the effects of novel antimicrobial agents on the normal functioning of the intestinal microbiota. The articles, written in English, were recovered from PubMed, ScienceDirect, Web of Science, Google Scholar, and DOAJ, as well as from manual searches using a reference list. “Microbiota”, “Intestinal Microbiota”, “Eubiotic Microbiota”, “Ecological Impact”, “Antimicrobial Agents,”, “Antibiotics”, “Dysbiosis”, “Gut Microbiota”, and “Probiotics” were the search terms used to retrieve the articles. The PRISMA 2009 checklist was applied for article search strategy, article selection, data extraction, and result reporting for the review process. A total of eight original research articles were included from a total of 379 articles obtained in different search strategies. The eight new antimicrobial agents demonstrated significant impacts on the ecological balance of the human intestinal microbiota. Therefore, eubiosis is crucial in preventing the establishment of exogenous antimicrobial-resistant strains as well as their gene transfer.

**Systematic review registration:**

[PRISMA], identifier [2009].

## 1 Introduction

Under normal conditions, the normal human microbiota is relatively stable in each ecological habitat and provides a myriad of benefits to the host’s health. The normal microbiota acts as a barrier against colonization by potentially pathogenic microorganisms as well as the overgrowth of already present microorganisms such as yeasts (e.g., *Candida* spp.) or *Clostridioides difficile* in the intestinal tract through competition for nutrients and adhesion sites, and by producing metabolites [e.g., short-chain fatty acids (SCFAs)] and bacteriocins. Controlling the growth of opportunistic microorganisms is known as “colonization resistance,” and it is maintained not only by normal microbiota but also by several anatomical and physiological factors such as peristalsis of the intestinal tract and the secretion of saliva, sweat, and gastric and bile acids (1–3).

Delivery methods, age, miRNAs, the host’s intestinal secretory function, geographical variation, food and diet habits, physical stress, disease, and antibiotics can affect the normal microbiota composition and result in disruptions of the human microbiota’s normal function (4–14). The frequent use of antimicrobial drugs can have several negative effects on the normal microbiota found in different parts of the body. For example, the length and frequency of antibiotic use increase the chance of infection by *C. difficile* by causing the loss of specific microbial populations from the microbiota, which deregulates the production of antimicrobial peptides (e.g., bacteriocins) or metabolites against pathogen colonization (15). The emergence of resistance among bacteria in the normal microbiota and the distribution of resistant genes by vertical or horizontal (conjugation, transformation, or transduction) gene transfer in the microbial community can contribute to increased loads of pathogenic and drug-resistant microbes. Furthermore, disturbed normal microbiota reduces colonization resistance, resulting in an overgrowth of already present microorganisms or exogenous pathogens (16, 17).

Administration of antimicrobial drugs significantly disturb the ecological equilibrium of the normal microbiota located within the human body. The spectrum of the drug, the dose, the method of administration, the pharmacokinetic and pharmacodynamic characteristics, and the *in vivo* inactivation of the agent determine the extent of disturbance of antimicrobial agents in the normal human microbiota. The intestinal microbiota can be affected by the incomplete absorption of oral medications and the secretion of an antimicrobial agent by the vaginal or intestinal mucosa, bile, salivary glands, or eccrine or apocrine sweat glands. As a result, antibiotic-resistant microbiota and their genes could consequently become more prevalent in different parts of the body (17, 18).

Antimicrobial resistance is a major global public health problem that poses a challenge to the treatment of diseases such as skin and soft tissue infections, *C. difficile* infection (CDI), endocarditis, meningitis, pneumonia, tuberculosis, malaria, and AIDS (19–24) due to the use of broad- or narrow-spectrum and ecologically non-favorable agents. Since, changes in the composition of the microbiota (through loss of diversity or loss of specific taxonomic groups) due to therapeutic agents, result in microbial imbalances with increased susceptibility to different conditions and comorbidities, i.e., gastrointestinal (GI) infections, diabetes, obesity, liver disease, colon cancer, and inflammatory bowel disease (IBD). Therefore, during prescription, it is crucial that clinicians understand how antimicrobial drugs affect the ecology of the human microbiota. Furthermore, to mitigate this problem, novel antimicrobial agents with activity against drug-resistant pathogens are urgently needed due to the rise in resistance in many Gram-positive and Gram-negative pathogenic microbes, such as methicillin-resistant *Staphylococcus aureus* (MRSA), pan-drug-resistant Gram-negative bacteria, carbapenem-resistant *Enterobacteriaceae* (CRE), extended-spectrum beta-lactamase-producing *Enterobacteriaceae* (ESBL-E) (25), and other extreme drug-resistant or pan-drug-resistant organisms such as *Mycobacterium tuberculosis*, vancomycin-resistant *Enterococci* (VRE), highly infectious and fluoroquinolone-resistant *C. difficile*, multidrug-resistant *Streptococcus pneumoniae*, and *Neisseria gonorrhoeae* (26–32). Using probiotics is another significant alternative to minimize antibiotic-associated complications and comorbidities.

## 2 Methodology

Several research articles concerning the effects of novel antimicrobials on intestinal microbiota were extensively searched and collected from different databases. Many published articles were available separately, and a detailed review was essential to combine all results to draw a conclusion and avoid any information conflicts, ambiguities, or misunderstandings. The review, which aimed to highlight the type of novel agents, the dose, drug administration, the number of subjects, the drug’s impact on human intestinal microbiota, and the means of drug elimination, was conducted according to systematic reviews, as recommended by Moher et al. (33) The PRISMA 2009 (i.e., Preferred Reporting Items for Systematic Reviews and Meta-Analyses) guidelines and checklist were strictly followed to document this review.

### 2.1 Formulation of research questions and problems

This systematic review was guided by the following question: “What are the ecological impacts of novel antimicrobial agents on the human intestinal microbiota?” The problem was formulated while searching and assessing the impacts of antimicrobial agents on human health. Due to their diverse impacts, the study focused on examining the impact of new antimicrobials on the intestinal microbiota. This question created a further interest in examining whether novel antimicrobial agents simultaneously affect the resident microbiota and human health or if these agents alternatively affect the transient microbiota.

### 2.2 Search engine for research articles

**Figure 1:**
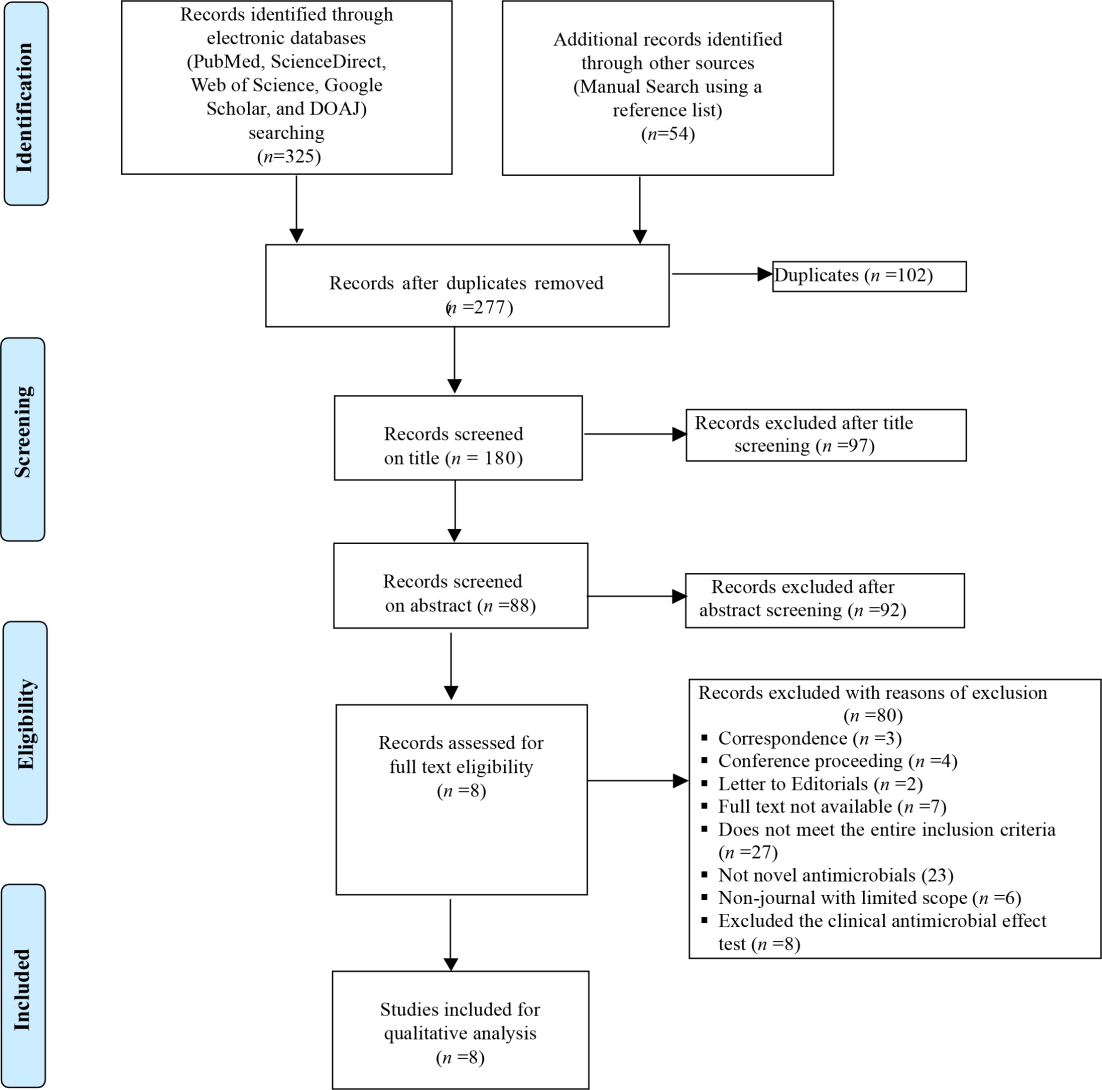
PRISMA 2009 flow diagram of eligible studies.

An extensive search of research articles was conducted in international electronic databases [PubMed, ScienceDirect, Web of Science, Google Scholar, and Directory of Open Access Journals (DOAJ)] and other sources (manual search using a reference list). The articles were searched using the following key terms and phrases taken from the title, abstract, and keywords in combination or separately using Boolean operators (“OR” or “AND”): “Microbiota”, “Intestinal Microbiota”, “Eubiotic Microbiota”, “Ecological Impact”, “Antimicrobial agents”, “Antibiotics”, “Dysbiosis”, “Gut Microbiota”, and “Probiotics”. The study was carried out from October 2022 to January 2023. The search process was presented in accordance with the PRISMA 2009 flow diagram (33) guidelines, together with the included and excluded items and reasons for exclusion (**Figure 1**).

### 2.3 Inclusion and exclusion criteria for included studies

In this systematic review, different studies conducted around the world were included. Articles collected through the searches were evaluated for inclusion in the systematic review based on the following criteria: (i) original articles on novel antimicrobial agents that address their impact on the ecological balance of the human intestinal microbiota; (ii) clinical studies; (iii) human studies only; (iv) only studies reported in English; (v) recent journals studied from 2006 to 2014; (vi) only intestinal microbiota; and (vii) articles published and available online. However, reports on the impacts of antimicrobials on other experimental animals, other microbiota, non-peer-reviewed articles, *in vitro* studies, other non-pharmaceutical agents, studies not published in English, review papers (comprehensive, scoping, systematic, meta-analysis, or other forms of review), duplicate publications (articles published in two journals with the same title, same first author, same study design, same sample size, and the same number of in-text citations or references), or extensions of analysis from original studies that were incompletely presented were excluded from the review process.

### 2.4 Data extraction

A data abstraction protocol was used to construct data from each of the included articles. The data extraction protocol consisted of the normal microbiota body sites; examples of the microbiota encountered and its functions are in **Table 1**. The type of new agents, the dose, drug administration, the method of drug receiving, the number of subjects, the drug’s impact on the human intestinal microbiota, the means of drug elimination, and references are in **Table 2**. Furthermore, type of patient, age, the type of probiotic species, probiotic dose (CFU) on a daily, probiotic therapy duration, and references are in **Table 3**. The selection of all

**Table 1:** Normal human microbiota encountered at various body sites, examples, and functions.

Normal microbiota body sites	Examples	Functions
Conjunctiva	Coagulase-negative staphylococci, *Haemophilus* spp., *S. aureus*, and various species of streptococci	Maintaining ocular homeostasis by using various mechanisms. For example, by producing more “lysozyme” they protect the eye from pathogens.
Nose	Coagulase-negative staphylococci, viridans streptococci, *S. aureus*, *Neisseria* spp., *Haemophilus* spp., and *S*. *pneumoniae*	They can combat opportunistic pathogen colonization through limited resources such as nutrients and space, and they can even create toxins that directly inhibit or destroy competing microbes.
Ear	Coagulase-negative staphylococci, *Corynebacterium* genus non-patogenic (Diphtheroids), *Pseudomonas* spp., and occasionally Enterobacteriaceae	Contribute to the natural antibacterial properties and aid the body in preventing ear infections.
Mouth and oropharynx	Viridans streptococci, coagulase-negative staphylococci, *Veillonella* spp., *Fusobacterium* spp., *Treponema* spp., *Porphyromonas* spp., *Prevotella* spp., *Neisseria* spp., *Branhamella catarrhalis*, *S*. *pneumoniae*, non-group A Beta- hemolytic streptococci, *Candida* spp., *Haemophilus* spp., *Corynebacterium* genus non-patogenic (Diphtheroids), *Actinomyces* spp., *Eikenella corrodens*, and *S. aureus*	They are important for the digestion of food through enzymatic mechanisms.
Skin	Coagulase-negative staphylococci, *Corynebacterium* genus non-patogenic (Diphtheroids), *S. aureus*, various species of streptococci, *Bacillus* spp., *Malassezia furfur*, *Candida* spp., and occasionally *Mycobacterium* spp.	By creating antimicrobial compounds (e.g., by making the skin surface slightly acidic and hyperosmotic environment), it prevents transient microbe invasion and other outcompeting microbes that land on the skin’s surface. They also produce fatty acids in the skin that inhibit pathogenic microbes.
Stomach	*Streptococcus* spp., *Staphylococcus* spp., Lactobacillaceae, *Peptostreptococcus* spp., *Helicobacter pylori*	They are important in the host’s nutrient metabolism, xenobiotic and drug metabolism, preservation of the gut mucosal barrier’s structural integrity, immunomodulation, and protection of the stomach against various pathogens.
Small intestine	Lactobacillaceae, *Bacteroides* spp., *Clostridium* spp., *Mycobacterium* spp., *Enterococci*, and Enterobacteriaceae	Provide synthesized vitamins to the host, such as biotin (vitamin B7) and folate (vitamin B9). Prevents the pathogenic microbes by making the environment alkaline.
Large intestine	*Bacteroides* spp., *Fusobacterium* spp., *Clostridium* spp., *Peptostreptococcus* spp., *Escherichia coli*, *Klebsiella* spp., *Proteus* spp., Lactobacillaceae, *Enterococci*, various species of streptococci, *Pseudomonas* spp., *Acinetobacter* spp., coagulase-negative staphylococci, *S. aureus*, *Mycobacterium* spp., and *Actinomyces* spp.	Participate in the formation of vitamin K, antibiotics, ammonia, bile acid conversion, and other fermentation by-products that interfere with the survival or proliferation of intestinal pathogens. Some of the normal anaerobic microbiota maintains the environment, making the intestine inhospitable to aerobic pathogens.
Urethra	Coagulase-negative staphylococci, *Corynebacterium* genus non-patogenic (Diphtheroids), various species of streptococci, *Mycobacterium* spp., *Bacteroides* spp. *Fusobacterium* spp., and *Peptostreptococcus* spp.	Play a crucial protective role in preventing uropathogens from adhering to the surface of uroepithelial cells.
Vagina	Lactobacillaceae, *Peptostreptococcus* spp., *Corynebacterium* genus non-patogenic (Diphtheroids), various species of streptococci, *Clostridium* spp., *Bacteroides* spp., *Candida* spp., and *Gardnerella vaginalis*	They contribute to host defenses against acid-intolerant and other potential vaginal pathogens by lowering the vaginal pH from approximately 4.4 to 4.6.

**Table 2:** The effect of novel antimicrobial agents on the ecological balance of human intestinal microbiota.

Agent	Dose [mg/day (24h)]	Drug administration	Method of drug receiving	Number of subjects	Impact on human intestinal microbiota	Drug on feces	References
Ceftobiprole	500 × 3	7/Parenterally administered	Intravenous infusion	12	Enteric bacteria (*E. coli*), *Enterococci*, *Bifidobacterium* genus, Lactobacillaceae, *Clostridia* spp., *Bacteroides* spp., and *Candida albicans*	No measurable concentrations	(34)
Ceftaroline	600 × 2	7/Parenterally administered	Intravenous infusion	12	*E. coli*, *Bifidobacterium* genus, and Lactobacillaceae	No measurable concentrations	(35)
Telavancin	10mg/kg × 1	7/Parenterally administered	Intravenous infusion	13	*Enterobacteriaceae*, *Enterococci*, *C*. *albicans*, *Bifidobacterium* genus, Lactobacillaceae, *Clostridia* spp., and *Bacteroides* spp.	No measurable (<1%) concentrations	(36)
Dalbavancin	1g	1/Parenterally administered	Intravenous infusion	12	*Enterococci* and *E. coli*	6.8 and 73.4 mg/kg on day 5 and 7.4e26.4 mg/kg on day 14	(37)
Tigecycline	50–100 × 2	10/Parenterally administered	Intravenous infusion	13	Oropharyngeal microbiota	3.0 and 14.1 mg/kg on day 8	(38)
Fidaxomicin	50–200 × 2	10/Perorally administered	Orally	23	Enterobacteriaceae, *Enterococci*, *Bifidobacterium* genus, *Lactobacillaceae*, *Clostridia* spp., and *Bacteroides* spp.	0.442 and 0.430 mg/kg on day 10	(39)
MCB3837	6mg/kg × 1	5/Parenterally administered	Intravenous infusion	12	*Enterococci*, *Bifidobacterium* genus, *Lactobacillaceae*, and *Clostridia* spp.	16.5 mg/kg and 98.9 to 226.3 mg/kg on day 2 and 5, respectively	(40)
Doxycycline	40 mg × 1	112/Perorally administered	Orally	17	*Enterococci* and *E. coli*	0–3.71 mg/kg and 0–4.10 mg/kg on 4- and 16-week visit, respectively	(41)

**Table 3:** The role of probiotics in decreasing antibiotic-associated diarrhea impacts among in- and out-patients.

Type of patient	Age (years)	Type of probiotic species	Probiotic dose (CFU) on a daily	Probiotic therapy duration	Reference
Inpatient	>18	■*B. longum* ■*L. acidophilus* ■*E*. *faecalis*	48×10^9^ or 24×10^9^	14 days	(42)
Inpatient	>18	■*L*. *casei* Shirota (Yakult)	6.5×10^9^	Duration of antibiotic+7 days	(43)
Inpatient	>65	■*L*. *casei* Shirota (Yakult)	13×10^9^	Duration of admission	(44)
Inpatient	>18	■*L*. *rhamnosus* R0011+*L. acidophilus* R0052	4×10^9^	14 days	(45)
Inpatient	>18	■*L*. *rhamnosus* GG	20×10^9^	14 days	(46)
Inpatient	>18	■*L. acidophilus* La-5 ■*L*. *casei* Lc-01 ■*B*. *lactis* Bb-12	17–23×10^9^	Duration of antibiotic+5 days	(47)
Inpatient	≥55	■L. casei DN114001 ■*L. delbrueckii* subspecies bulgaricus ■*S. thermophilus*	20.4×10^9^	Duration of antibiotic+7 days	(48)
Inpatient	>18	■*L. acidophilus*	60×10^9^	Duration of antibiotic+14 days	(49)
Inpatient	>18	■*L. acidophilus*+*L*. *casei*	50×10^9^	Duration of antibiotic+5 days	(50)
Inpatient	>18	■*B. breve* ■*B. longum* ■*B*. *infants* ■*L. acidophilus* ■*L. plantarum* ■*L. paracasei* ■*L. delbrueckii* subspecies bulgaricus ■*S. thermophilus*	900×10^9^	Duration of antibiotic+7 days	(51)
Inpatient	30–70	■*L. acidophilus* NCFM (ATCC700396) ■*L. paracasei* Lpc-37 (ATCC SD5275) ■*B*. *lactis* Bi-07 (ATCC SD5220) ■*B*. *lactis* Bl-04 (ATCC SD5219)	17×10^9^, 4.17×10^9^	Duration of antibiotic+7 days	(52)
Inpatient	>16	■*L. plantarum* 299v	10×10^9^	Duration of antibiotic+7 days	(53)
Inpatient	>18	■*L. acidophilus* (gasseri) ■*L*. *helveticus* (bulgaricus) (Lactinex)	4×10^9^	5 days	(54)
Inpatient	50–70	■*L. acidophilus* CL1285 ■*L*. *casei* LBC80R	100×10^9^, 50×10^9^	Duration of antibiotic+7 days	(55)
Inpatient	40–77	■*S*. *boulardii*	36×10^9^	Duration of antibiotic+7 days	(56)
Inpatient	>18	■*L*. *reuteri* ATCC 55,730 (BioGaia Biologics, Sweden)	0.2×10^9^	28 days	(57)
Inpatient	>18	■*L. acidophilus* ■*L*. *bulgaricus* ■*S. thermophilus*	0.002×10^9^	8 days	(58)
Inpatient	54–85	■*L. acidophilus* ■*L*. *casei* (Bio- K+CL1285, Bio- K+International, Canada)	50×10^9^	Duration of antibiotic	(59)
Inpatient	≥65	■*L. acidophilus* ■*B*. *bifidum*	60×10^9^	21 days	(60)
Outpatient	>15	■*S*. *boulardii*	10.2×10^9^	12 days	(61)
Outpatient	≥45	■*B*. *subtilis* 3 and *B*. *licheniformis* 31 ■*B*. *licheniformis*	4×10^9^	Duration of antibiotic+7 days	(62)

recovered articles was carried out step by step by Abayeneh Girma, and finally the extracted data were combined and clearly presented in the table with key information and findings. The period from 1 November to 30 December 2022 was used for study selection, quality evaluation, and data extraction.

### 2.5 Quality assessment of each included study

For a systematic review, the PRISMA 2009 checklist (33) is the best tool to assess and examine the validity, reliability, and presentation quality of all extracted data from each included article. The choice and evaluation of the quality were performed by Abayeneh Girma as per the flow diagram presented in **Figure 1**. The articles were added after carefully checking their quality.

## 3 Results

In total, 379 articles on the ecological impacts of novel antimicrobial agents on human intestinal microbiota were recovered from across the world. One hundred and two of these articles were excluded due to duplicates. Of the remaining 277 articles, 97 were excluded after screening the titles. Of the remaining 180 articles, 92 were also excluded after abstract selection. Of the 88 articles, 80 were further excluded after observation and review due to the inclusion and exclusion criteria used. Therefore, only eight of the studies met the eligibility criteria and were included in the final systematic review (**Figure 1**). Of the included articles, six investigated the parenteral type of administration of the agents, while the remaining two articles tested by peroral type of administration (**Table 2**). Six studies used intravenous infusion and two studies used oral administration (**Table 2**). One of the included articles evaluated the impacts of antimicrobial agents on 23 subjects, and the other seven articles assessed fewer than 20 subjects (**Table 2**). Five of the included articles showed the presence of drugs on faces during excretion on different days, while the remaining three articles reported no measurable concentrations of drugs on feces (**Table 2**).

## 4 Human microbiota

**Figure 2:**
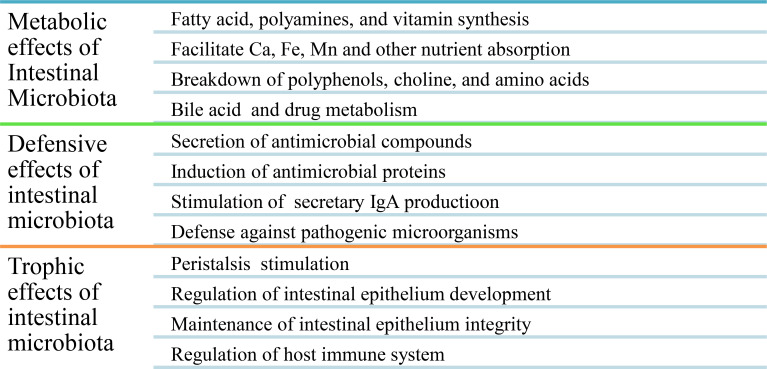
Summary of the beneficial effects of eubiotic intestinal microbiota.

The normal human microbiota (**Table 1**), also known as the normal microbiota, the indigenous microbial population, or microbiota, is a mixture of microorganisms that regularly inhabit any site in the human body and contribute to the regulation of a host’s health. The internal organs and systems, including the spleen, pancreas, liver, bladder, central nervous system, and blood, are sterile apart from occasional transient intruders. A healthy newborn was previously reported as being practically sterile when it is born but quick to acquire the typical microbiota from food and the environment, even from other people (63, 64). Recent findings, however, revealed that bacteria are present in the placenta, umbilical cord blood, fetal membranes, and amniotic fluid of healthy neonates who show no symptoms of illness or inflammation. The meconium (first stools of newborns) of premature infants with healthy mothers contains a particular microbiome, with the main phyla being Bacillota and a predominance of *Staphylococcus* spp., while the Pseudomonadota phyla are present in species such as *Escherichia coli*, *Klebsiella pneumoniae*, and *Serratia marcescens* (4, 63, 65, 66). In a healthy person, these microbes are generally harmless and even helpful through metabolic, defensive, and trophic functions (**Figure 2**). For all humans, the species found in the normal microbiota cannot be precisely defined because they differ from person to person due to physiological variances, dietary preferences, age, and geographical habitat (67).

## 5 Classification of the microbiota

Normal microbiota are clearly classified as resident (they permanently reside in a given body site, e.g., the skin, because they do not completely flush off from a given anatomical site and they have the potential to re-establish after disturbance, and this type of microbe does not associate with the transmission of disease because they are totally non-pathogenic) or transient (they reside temporarily, cannot re-establish after destruction, can flush off the body site, can be pathogenic or non-pathogenic, and this group is highly associated with disease transmission since they become opportunistic pathogen due to dysbiosis).

## 6 Beneficial effects of eubiotic microbiota

Eubiotic microbiota are important in the synthesis of different vitamins (e.g., vitamins K, B7, and B9), for the digestion and absorption of different nutrients, for improving lactose use in people with lactose intolerance and serum cholesterol concentration, for producing volatile fatty acids (e.g., acetic, propionic, and butyric acids) by anaerobic bacteria that are toxic to the growth of enterobacteria in the gut, for preventing the proliferation of disease-causing microbes by lowering the pH of the environment, for producing organic acids and substances (e.g., bacteriocins) that discourage colonization by exogenous microorganisms, for competition with pathogens for nutrients and space (attachment sites), for the inactivation of microbial toxins (e.g., bacteria or fungi) or metabolites, for stimulating non-specific immunity, and for increasing the brain development and behavior of the host (67) (**Table 1**). Generally, metabolic, defensive, and trophic effects are the main beneficial effects of eubiotic intestinal microbiota (15) (**Figure 2**).

## 7 The effect of antimicrobial agents on the intestinal microbiota and its resistance mechanisms

**Figure 3:**
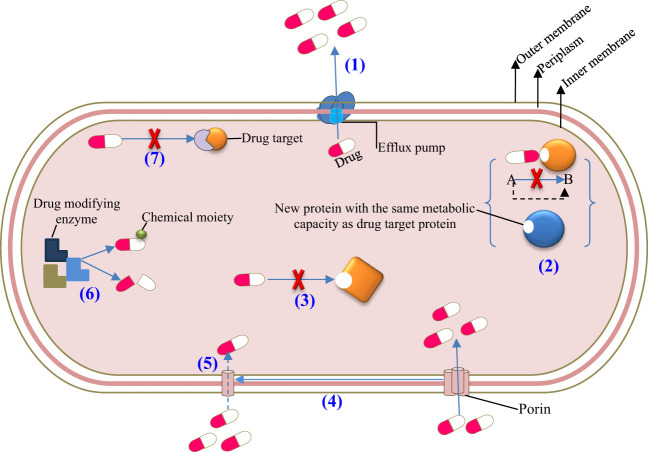
Resistance mechanisms of microbiota after frequent exposure to antimicrobial agents. (1) Active efflux, (2) Target bypass, (3) Target site modification, (4) Downregulation, (5) Decreased influx, (6) Drug inactivation, and (7) Target protection.

Antimicrobial agents have a great impact on the human body and its microbiota. For example, the phyla Firmicutes, Bacteroidetes, Actinobacteria, and Proteobacteria mainly dominate the human intestinal microbiota; however, they are highly sensitive to the agent’s deleterious effect. Administration of antimicrobial drugs is the most common and substantial cause of changes in the normal gut microbiota. The antimicrobial agents’ effect on the ecological balance of the human intestinal microbiota has direct and indirect impacts on the health of the host. When the normal microbiota of the intestine is reduced in species diversity during therapy, the resistance to colonization is significantly decreased, leading to the following outcomes: (i) The resistance of microorganisms to administered antimicrobial agents allows them to proliferate in large concentrations in the intestine. (ii) Allows the overgrowth of exogenous bacteria, yeast, and other microbes. (iii) Pathogenic microorganisms that are resistant to antimicrobial agents and colonies within the intestine can spread to different parts of the host. (iv) Overgrowth of pathogenic microorganisms in the intestine encourages the transfer and spread of resistant organisms and their genes either vertically or horizontally (**Figure 3**). (v) A lower contamination threshold dose results from a decrease in resistance to colonization (68). (vi) Reduces the diversity of species in the intestinal microbiota. (vii) Alters metabolic activity. (viii) Causes antibiotic-associated complications and comorbidities such as obesity, asthma, allergies, and IBD, especially in children (15, 69–71).

Furthermore, the frequency and duration of antimicrobial treatment affect the intestinal microbiota and make the area of the resistome favorable for antimicrobial-resistant pathogens and their genes. Resistomes are intestinal microbes that have numerous genes for antibiotic resistance. The resident resistome (commensal bacteria containing antibiotic-resistant genes) and the transitory resistome (antibiotic-resistant genes carried by bacteria periodically) are the two different types of resistomes found in the intestinal microbiota. The transient resistome has the potential to become a long-term component of the microbiota or pass on its resistance gene to commensal bacteria. Therefore, it is of high interest to determine the gut microbiota of people who have the antibiotic-resistant gene and understand how the gene can spread among various commensal organisms and opportunistic pathogens (**Figure 3**). Generally, the alteration of the intestinal microbiota composition due to antimicrobial agents results in dysbiosis or disease and affects the host’s health (71–74). Some of the antimicrobial agents that have been recently approved by the US Food and Drug Administration (FDA) for clinical use are listed in **Table 2**, and their brief effects on the intestinal microbiota are presented as follows:

### 7.1 Ceftobiprole

**Figure 4:**
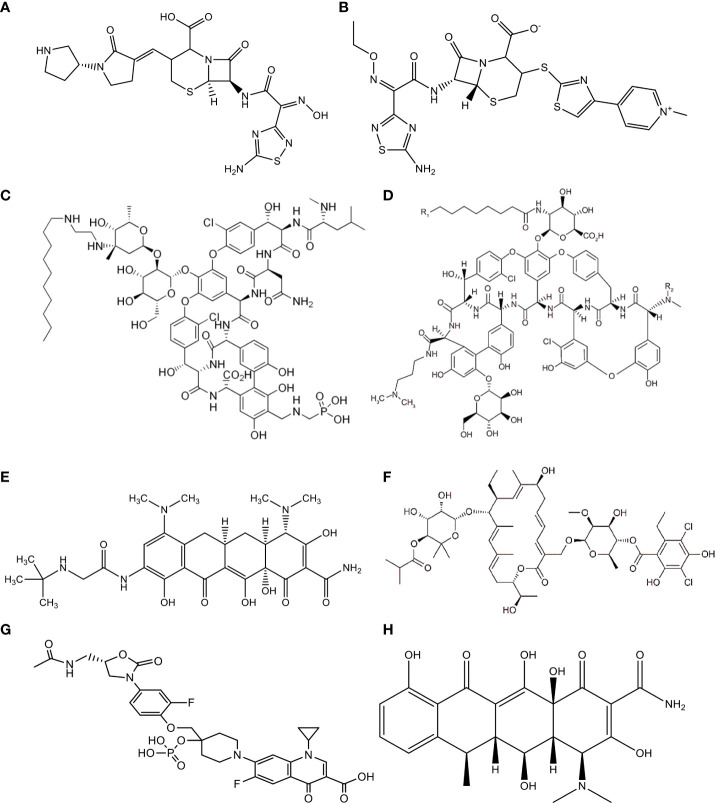
Chemical Structure of **(A)** Ceftobiprole, **(B)** Ceftaroline, **(C)** Telavancin, **(D)** Dalbavancin, **(E)** Tigecycline, **(F)** Fidaxomicin, **(G)** MCB3837, **(H)** Doxycycline.

The new broad-spectrum cephem antibiotic known as “ceftobiprole” (**Figure 4A** and **Table 2**) is an effective antimicrobial agent against Gram-negative bacteria such as Enterobacteriaceae and *Pseudomonas* spp., as well as methicillin-resistant *Staphylococcus aureus* (MRSA) and vancomycin-resistant *Enterococcus faecalis* (75–77). Ceftobiprole is a preliminary cephalosporin that shows clinical efficacy in patients with MRSA-causing infections. It is a potential antibacterial drug for the treatment of pneumonia and severe skin infections. According to different reports, it is a well-accepted antimicrobial agent with good safety for the host and is excreted in urine. Furthermore, according to the reports of Bäckström et al. (34), this agent had no remarkable ecological effect on the human microbiota of the intestine. *Bacteroides fragilis* and *Prevotella* spp. are resistant to ceftobiprole, which implies that the agent is secreted by the intestinal mucosa or bile, affecting the normal functioning of the intestinal microbiota and causing resistance to antibiotics.

### 7.2 Ceftaroline

A new semisynthetic parenteral cephalosporin antimicrobial agent with broad-spectrum action is called “ceftaroline” (**Figure 4B** and **Table 2**). Unlike other cephalosporins, ceftaroline continues to be active against MRSA, penicillin-resistant pneumococci, and Gram-negative infections. It is a potential antibacterial drug that can be used to treat complex skin and skin structure infections, as well as pneumonia caused by a bacterial community infection (78). According to reports, ceftaroline is a well-accepted and safe antimicrobial agent, and renal excretion is the means of drug elimination (79–81). However, *B*. *fragilis* and *Prevotella* species are resistant to this antibiotic. This might be due to the secretion of ceftaroline by intestinal mucosa or bile, which leads to antibiotic resistance. Furthermore, Panagiotidis et al. (35) reported that this antibiotic had a minor impact on the normal function of the human intestinal microbiota.

### 7.3 Telavancin

A novel and narrow-spectrum lipoglycopeptide antibiotic used for the treatment of Gram-positive aerobic and anaerobic bacterial pathogens is known as “telavancin” (**Figure 4C** and **Table 2**). Inhibition of bacterial cell wall synthesis and disruption of the plasma membrane are the main mechanisms of action of this antibiotic (82, 83). According to Saravolatz et al. (84), Stryjewski et al. (85), Rubinstein et al. (86), and Finegold et al. (87), telavancin is crucial for the treatment of severe skin and skin structure infections, bacterial infections, and pneumonia acquired in hospitals that are caused by MRSA, methicillin-susceptible *Staphylococcus aureus* (MSSA), *S*. *pneumoniae*, *S*. *pyogenes*, *S*. *agalactiae*, *S*. *anginosus*, *S*. *intermedius*, or *S*. *constellatus*, as well as vancomycin-susceptible *E*. *faecalis*. This antibiotic had higher activity against MRSA and *Clostridium* spp. than other antibiotics such as vancomycin. However, telavancin is not effective against aerobic and anaerobic bacteria due to the lipopolysaccharide found in these organisms, which makes the target inaccessible to this antimicrobial agent. Furthermore, according to different reports, telavancin is a safe and well-tolerated antibiotic, with 60% to 70% of the drug eliminated by renal excretion and <1% in feces (36, 88, 89).

### 7.4 Dalbavancin

A novel and narrow-spectrum lipoglycopeptide antibiotic effective in the laboratory against most Gram-positive bacterial pathogens is called “dalbavancin” (**Figure 4D** and **Table 2** **)**; these pathogens include staphylococci, streptococci, *Enterococci*, corynebacteria, and anaerobic bacteria (90, 91). Its *in vitro* activity is also observed in MSSA, MRSA, and coagulase-negative staphylococci (CNS) such as *S*. *epidermidis*, *S*. *haemolyticus*, and others (30). Furthermore, it is highly effective against *S*. *pneumoniae* (either susceptible or resistant to penicillin), vancomycin-susceptible bacteria, and some classes of resistant *Enterococci* (30, 31). Different clinical studies around the world have confirmed that administration of this antibiotic once a week is highly effective in the treatment of Gram-positive bacterial pathogens. This agent is reported as a well-tolerated and safe antibiotic with no major ecological impact on the intestinal microbiota. The drug is excreted through both urine and feces (37, 92–94).

### 7.5 Tigecycline

A broad-spectrum and novel analog of minocycline antibiotic analogs that demonstrates activity against aerobic and anaerobic Gram-positive and Gram-negative pathogenic bacteria, including antimicrobial-resistant bacterial strains, is known as “tigecycline” (**Figure 4E** and **Table 2**) (95–99). This antibiotic is crucial for the treatment of severe skin and skin structure infections, as well as intra-abdominal infections. The reports also suggest that this antibiotic is tolerable and safe for the host. This drug is excreted through biliary elimination and, therefore, has a temporary impact on the ecological balance of the human intestinal microbiota (*Enterococci*, *E. coli*, Lactobacillaceae, and *Bifidobacterium* genus), apart from the oropharyngeal microbiota, due to the broad spectrum and high concentrations of tigecycline in the intestine (38, 100–102).

### 7.6 Fidaxomicin

A novel, narrow-spectrum and 18-membered-ring macrocyclic bactericidal agent developed primarily to treat CDI is called “fidaxomicin” (**Figure 4F** and **Table 2**). *In vitro*, this antibiotic was highly effective against CDIs compared with the other, poorly absorbed, oral antibiotic known as “vancomycin” (103, 104). Fidaxomicin is less effective against Gram-positive spore formation, such as *Propionibacterium*, Lactobacillaceae and *Peptostreptococci*, as well as being ineffective against aerobic and anaerobic Gram-negative bacilli such as Enterobacteriaceae, *Pseudomonas* spp., *Campylobacter* spp., *Helicobacter* spp., *Haemophilus* spp., *Bacteroides* spp., *Fusobacterium*, *Porphyromonas* spp., *Prevotella* spp., and *Veillonella* spp. This antibiotic prevents transcription through the inhibition of the RNA polymerase enzyme. According to different studies, as compared with vancomycin, it had a low ecological impact on the human intestinal microbiota. *B*. *fragilis* also resists this drug (39, 105).

### 7.7 MCB3837

A novel, fluoroquinolone-oxazolidinone, narrow-spectrum, and water-soluble synthetic antibiotic that targets only Gram-positive bacteria is called “MCB3837” (**Figure 4G** and **Table 2**), which belongs to a group of medicines known as “quinolone antibiotics”. After parenteral administration *in vivo*, oxaquin is rapidly converted to its active form called “MCB3681”. It is a bactericidal agent developed primarily to treat CDI (106–108). In addition, it is used to treat bacterial infections such as severe skin and soft tissue infections, genital tract infections, acute exacerbation of chronic obstructive pulmonary disease, community-acquired pneumonia (CAP), bronchitis, and mild to moderate pelvic inflammatory disease. The mechanism of action of MCB3681 is to bind to a DNA gyrase enzyme and block the DNA replication of the pathogen. The reports also suggest that this antibiotic is considered ecologically favorable for the host. This drug has a pronounced impact on the ecological balance of the human intestinal microbiota (*Enterococci*, Lactobacillaceae, Clostridia, and *Bifidobacterium* genus), apart from skin, nasal and oropharyngeal microbiota due to its spectrum, pharmacokinetic properties, and high concentrations of MCB3681 in the intestine without resistance development (40).

### 7.8 Doxycycline

A novel, broad-spectrum, and semi-synthetic antibiotic that belongs to the class “tetracyclines” and is effective against, aerobic, and anaerobic Gram positive and Gram-negative bacteria, spirochetes, and mycoplasma is called “Doxycycline” (**Figure 4H** and **Table 2**). It is primarily developed for the treatment of anthrax caused by *Bacillus anthracis*. Inhibition of protein synthesis by allosterically binding to the 30S bacterial ribosomal unit is the mechanism of action of doxycycline. It has a minor ecological effect on the oropharyngeal and intestinal microbiota. The drug is excreted through both urine and feces. Furthermore, the agent is secreted by the intestinal mucosa or bile and affects the normal functioning of the intestinal microbiota (*Enterococci* and *E. coli*) through inflammatory effects and causes resistance to antibiotics (41, 109, 110).

## 8 The effect of diet and medicines on microbiota diversity among Western and non-Western countries

**Figure 5:**
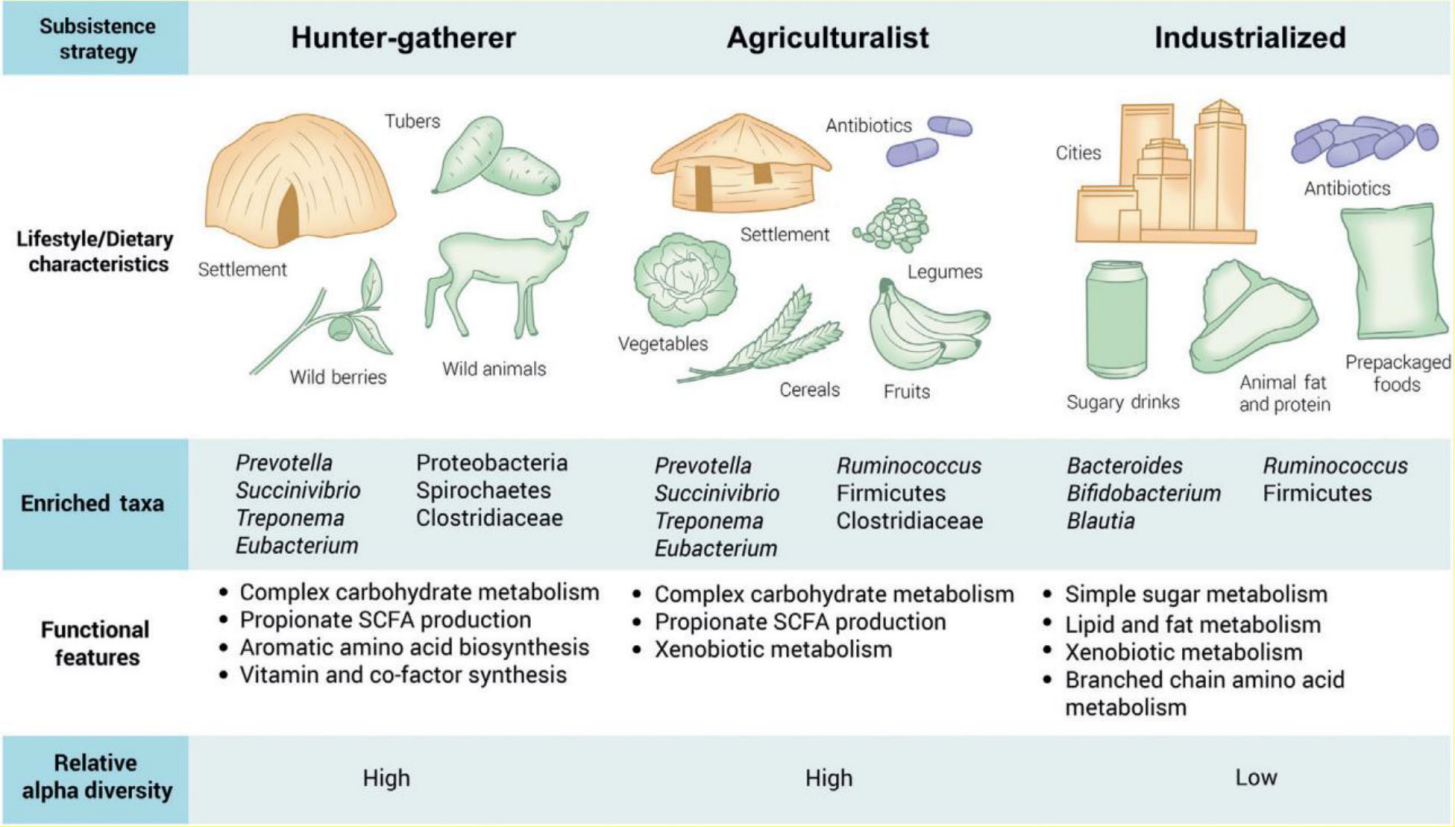
Effects of diet and medicines on microbiota diversity among Western and non-Western countries. Adapted from (111) with permission from Elsevier Ltd.

It is generally known that a long-term diet affects the microbiome’s taxonomic composition and functional characteristics (**Figure 5**). The inverse association between *Prevotella* and *Bacteroides*, two members of the Bacteroidetes phylum, is a recurring pattern. *Prevotella* is associated with a diet high in plant-based foods, which is predominant in non-industrialized people, but *Bacteroides* is associated with a larger intake of animal fats and proteins, which is typical in industrialized populations (112–115).

With regard to antibiotic utilization, hunter-gatherer nations did not use it, while agriculturalists utilized fewer antibiotics than industrialized countries, which utilized more drugs either separately or in combination with food or water (111). As a result, according to Brewster et al. (111), we can conclude that in Western or developed countries, the intestinal microbiota diversity and function are more highly affected by medicines and diet than in non-Western or developing countries. Similarly, Mosca et al. (116) reported that dysbiosis and a lack of microbial diversity in the gut microbiota are linked to the majority of human diseases that strike Westernized nations. This is because the widespread use of antibiotics and other environmental triggers in Western lifestyles means there may be fewer bacterial predators, which could result in less microbial diversity in the human gut. Furthermore, according to Nasiri et al. (117), the protective gut microbiota is significantly reduced in patients taking broad-spectrum antibiotics, which promote the growth of *Clostridioides difficile*, which can be present in certain people at low levels.

## 9 The role of probiotics against the side effects of antimicrobial agents

Probiotics are living microorganisms that, when taken in substantial amounts, confer host health. Lactobacillaceae, *Bifidobacteria*, and yeasts (e.g., *Saccharomyces boulardii*) are the most known, commercially exploited, and important probiotic organisms to human health. They increase the growth of beneficial intestinal microbiota, compete with intestinal pathogenic microbes (e.g., *E. coli*, *C. difficile*), produce antimicrobials (e.g., bacteriocins) that kill intestinal pathogens, and induce the immune response of the host (e.g., production of “reuterin” *Limosilactobacillus reuteri*) (**Table 3**). Furthermore, it is important in the control of antibiotic-associated diarrhea (e.g., European Societies for Paediatric Gastroenterology, Hepatology, and Nutrition guidelines recommend the use of *Lacticaseibacillus rhamnosus* and *S*. *boulardii* probiotic therapy to prevent the harmful effects of antibiotic-associated diarrhea). Furthermore, the Lactobacillaceae and *Bifidobacteria* probiotics in combination with *H. pylori* eradication therapy are more effective at eradicating *H. pylori* in the stomach than therapy without probiotics (15, 118–126).

## 10 Future approved novel antimicrobial agents under different developmental stages

**Figure 6:**
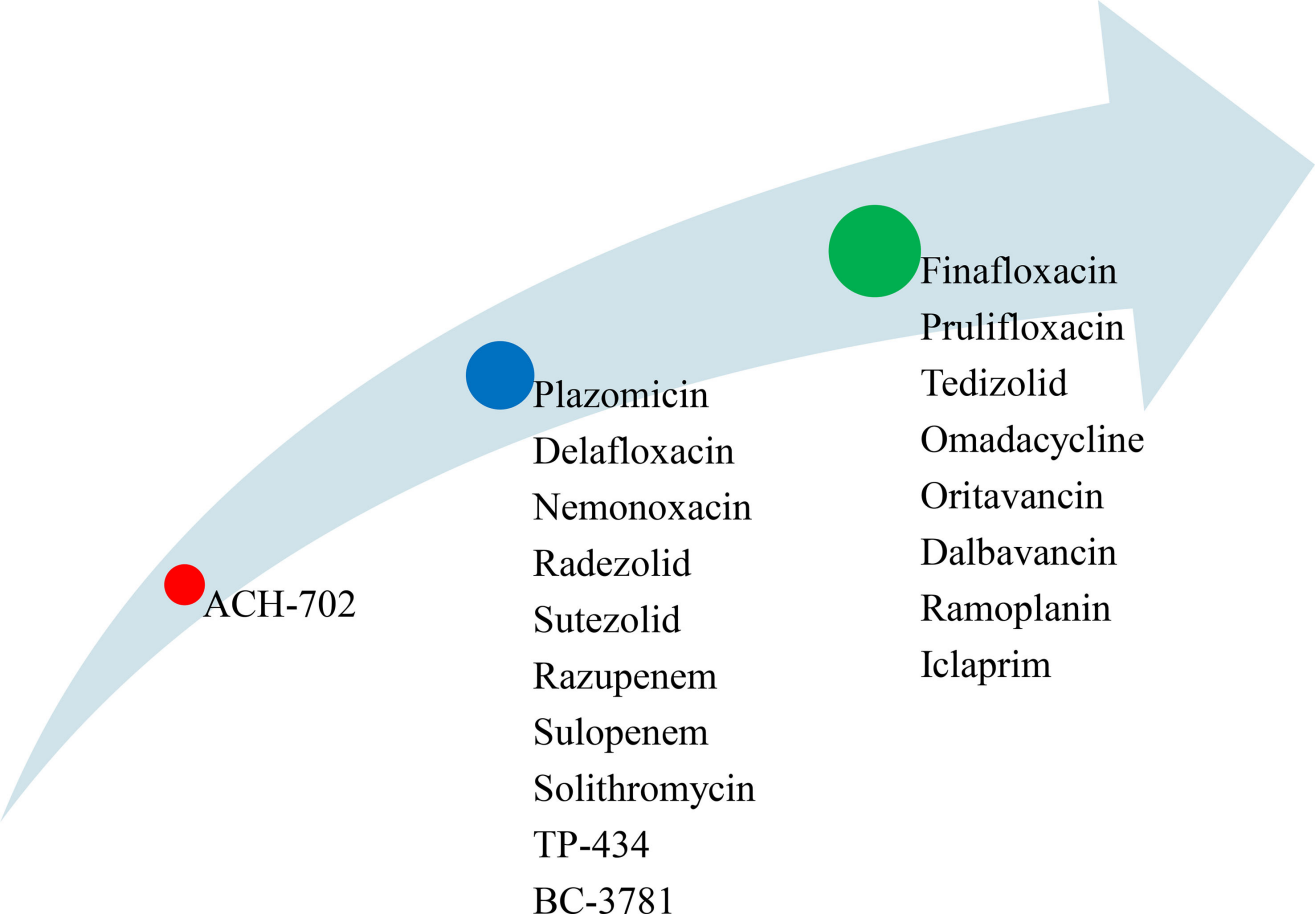
List of novel antimicrobial agents under different development stages (red=preclinical stage, blue= second phase, and green = third phase).

Currently, novel antimicrobial agents are highly needed to reduce the increasing number of multidrug resistant (MDR) microbial pathogens and the impacts of dysbiosis on the intestinal microbiota. ACH-702 is a preclinical stage antimicrobial agent active against both Gram positive and Gram-negative bacterial pathogens including MRSA and *M. tuberculosis* (**Figure 6**). Plazomicin (active against Gram-positive and Gram-negative bacteria), delafloxacin (for the treatment of infections in low pH environments such as the skin and vaginal and urinary tracts), nemonoxacin (for the treatment of CAP and diabetic foot infections), radezolid (for the treatment of complicated skin and soft tissue infections, and CAP), sutezolid (for the treatment of extensive drug-resistant tuberculosis), razupenem (for the treatment of complicated skin and soft-tissue infections caused by MRSA, VRE), sulopenem (for the treatment of skin and soft-tissues infections, and CAP caused by various aerobic Gram-positive and Gram-negative organisms as well as anaerobes), solithromycin (for the treatment of CAP and other Gram-positive infections), TP-434 (active against Gram-positive and Gram-negative pathogens including MRSA, *Streptococcus pyogenes*, and *E. coli*), and BC-3781 (to treat serious skin and skin structure infections) are collectively grouped under the second phase of the developmental stage (**Figure 6**). Finally, finafloxacin (demonstrates activity against MRSA, VRE, anaerobes, and other drug-resistant strains), prulifloxacin (active against Gram-positive and Gram-negative bacteria causing urinary and respiratory tract infections), tedizolid (activates against Gram-positive bacterial pathogens, including linezolid-resistant strains), omadacycline (for the treatment of MRSA), oritavancin (for the treatment of Gram-positive bacterial pathogens including MRSA, VRSA, and VRE), dalbavancin (for the treatment of VRE), ramoplanin (for the treatment of local gastrointestinal infections caused by *C. difficile*), and iclaprim (for the treatment of complicated skin and soft-tissue infections caused by *S. aureus* and *S*. *pneumoniae*, *H*. *influenzae*, *Moraxella catarrhalis*, and *Legionella pneumophila*) are found under the third phase of the developmental stage (**Figure 6**) (127–138).

## 11 Conclusions and future perspectives

Antimicrobial agents can cause several adverse effects on the normal functioning of the human microbiota. Based on the spectrum of antibiotics, dose, administration route, pharmacokinetic and pharmacodynamic properties of antibiotics, and *in vivo* inactivation of the agent, the extent of disturbances can vary between clinical conditions, such as systemic infections in immunocompromised patients and antibiotic-associated diarrhea or colitis. Ceftobiprole, ceftaroline, telavancin, dalbavancin, tigecycline, fidaxomicin, MCB3681, and doxycycline have minor impacts on the ecological balance of the human intestinal microbiota. Generally, clinicians should consider the antibiotic interaction between the agent administered and the normal intestinal microbiota when they choose the agent to treat microbial infections. In the future, novel antimicrobial agents and probiotics are highly needed to reduce the increasing impact of MDR pathogens and dysbiosis on intestinal microbiota health.

## Author contributions

The author confirms being the sole contributor of this work and has approved it for publication.
